# Emergency endotracheal intubation in critically ill patients with COVID-19: management and clinical characteristics

**DOI:** 10.1007/s44254-023-00003-9

**Published:** 2023-03-13

**Authors:** Fuquan Fang, Jing Jin, Yongmin Pi, Shaohui Guo, Yuhong Li, Shengmei Zhu, Xianhui Kang

**Affiliations:** 1 Department of Anesthesiology, the First Affiliated Hospital, Zhejiang University School of Medicine, No. 79 Qingchun Road, Hangzhou, 310003 China; 2 Zhejiang Center for Clinical Laboratory, Zhejiang Provincial People’s Hospital, People’s Hospital of Hangzhou Medical College, Hangzhou, 310014 China; 3 Department of Anesthesiology, Shulan (Hangzhou) Hospital, Shulan International Medical College, Shuren University, Hangzhou, 310015 China

**Keywords:** COVID-19, Tracheal intubation, Airway management, Critical care

## Abstract

**Purposes:**

SARS-CoV-2 have become widespread worldwide since the outbreak. Respiratory function deteriorates rapidly in critically ill patients infected with SARS-CoV-2. Endotracheal intubation is an indispensable therapeutic measure during the development of the disease. This study was intended to describe the experience of endotracheal intubation from front-line anesthesiologists and clinical prognosis of patients infected with Coronavirus disease-19 (COVID-19).

**Methods:**

Fourteen critical patients infected with COVID-19 who underwent endotracheal intubation were included in this study. We collate and analyze the blood gas results before and after tracheal intubation of patients and clinical prognostic indicators such as length of stay and. mortality. The experience of anesthesiologists who intubated patients has also been recorded in detail.

**Results:**

Patients had a mean time of 10.6 days from initial symptoms to endotracheal intubation. Most intubated patients had one or more underlying conditions: hypertension (8, 57.14%), diabetes (5, 35.71%), and cardiovascular and cerebrovascular diseases (2, 14.29%). The oxygenation index increased significantly after intubation compared with before intubation (148.80 ± 42.25 vs 284.43 ± 60.17 *p* < 0.001). 85.72% of patients required extra-corporeal membrane oxygenation (ECMO) due to inability to maintain oxygen saturation with standard therapeutic measures. Two patients underwent lung transplantation because their lungs were essentially nonfunctional, and they recovered well after surgery. As of this writing, all patients were discharged after satisfactory recovery.

**Conclusions:**

Reasonable selection of intubation timing is particularly important. It is crucial to increase the patient's oxygen supply and reduce oxygen consumption as much as possible during endotracheal intubation. In addition, the personal protective measures of medical personnel participating in treatment should be scientific and standardized.

**Graphical Abstract:**

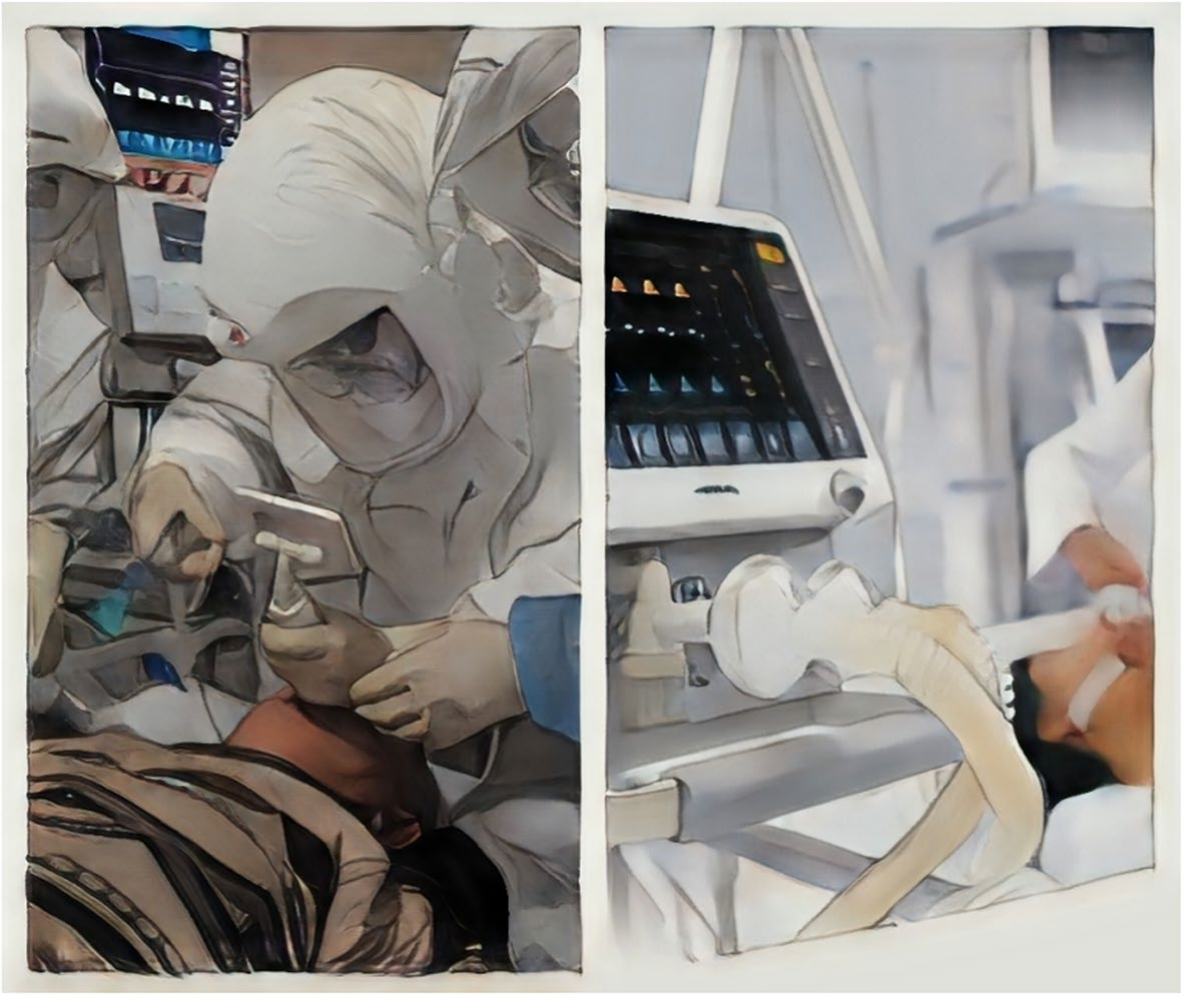

## Introduction

COVID-19 due to severe acute respiratory syndrome coronavirus 2 infection broke out in late 2019 and spread rapidly throughout the world [1, 2]. As of the time of this writing, there had been more than 100 million cases of infection and more than 2 million deaths worldwide. Studies have shown that among the COVID-19 patients in China, 14% of critically ill patients had severe dyspnea, manifested as respiratory rate ≥ 30 min^−1^, saturation ≤ 93%, oxygenation index < 300 mmHg, and/or pulmonary infiltration greater than 50%; 5% were critically ill patients, who experienced respiratory failure, sepsis, and multiple organ failure [3].

High-flow nasal cannula oxygen therapy and non-invasive positive pressure ventilation are used for the initial treatment of patients with COVID-19-associated respiratory failure [4, 5]. The World Health Organization and other institutions recommend avoiding its use in patients with continuous worsening hypoxemia, hemodynamic instability, and multiple organ failure [6, 7]. A study of 310 patients with acute respiratory distress syndrome (ARDS) showed that high-flow nasal cannula oxygen therapy did not significantly reduce the rate of endotracheal intubation [8]. Studies have reported that non-invasive positive pressure ventilation increases mortality in critically ill patients with ARDS [9–11]. This increase in mortality may be caused by a delay in the timing of endotracheal intubation. Therefore, endotracheal intubation is an essential treatment measure after respiratory deterioration in patients with severe COVID-19. Accurate grasp of the timing and criterion for endotracheal intubation is a challenge for anesthesiologists. At the same time, endotracheal intubation is one of the clinical procedures with the highest risk of close contact infection, and during the SARS epidemic in 2003, studies reported that endotracheal intubation was an important risk factor for healthcare providers to be infected through patients (odds ratio, 6.6) [12].

This study was intended to elaborate on the detailed process and preventive measures of endotracheal intubation for COVID-19 critically ill patients from front-line anesthesiologists in the first affiliated hospital, Zhejiang University school of medicine. The general clinical characteristics, important clinical treatment measures and related clinical outcomes of patients undergoing endotracheal intubation are also summarized.

## Methods

### Patient recruitment

This is an observational case series aiming to present COVID-19 patients that underwent intubation. Fourteen critically ill patients with COVID-19 who underwent endotracheal intubation were enrolled. Due to the small number of critically ill COVID-19 patients and the small sample size of this study, the conclusions of the paper should be treated with caution. Ethical approval was provided by the Clinical Research Ethical Committee of the First Affiliated Hospital, College of Medicine, Zhejiang University, Hangzhou, China (Chairperson Prof. Youming Li, Reference Number: IIT20200307A) on 19 July 2020. The trial was registered in Chinese Clinical Trial Registry (ChiCTR2000034852) and written informed consent was obtained. Fourteen patients diagnosed with COVID-19 and underwent endotracheal intubation during treatment in the hospital were enrolled.

### Data collection and study procedures

Demographic data and medical history were recorded. Saturation of pulse oximetry (SpO_2_) and blood pressure(BP) during endotracheal intubation were obtained from the anesthesiologist's operating records. Arterial blood gas detection results were obtained within 1 h before and 1 h after endotracheal intubation, and the difference of partial pressure of oxygen (PaO_2_) and partial pressure of carbon dioxide (PaCO_2_) before and after endotracheal intubation was compared. The time from the onset of symptoms to endotracheal intubation in COVID-19 patients was recorded. Other important treatments, such as Continuous Renal Replacement Therapy (CRRT), Extracorporeal Membrane Oxygenation(ECMO), and lung transplantation, were also recorded. The patients' survival was followed up by telephone 1 year later.

### Statistical analysis

Statistical analysis was performed using SPSS 23. Results for continuous data are expressed as means ± standard deviation (± SD). Group comparisons of numerical data were performed by Student’s t-test or Mann–Whitney test, as appropriate. *P* < 0.05 was considered statistically significant.

## Results

### Characteristics of COVID-19 patients treated with endotracheal intubation

A total of 105 patients diagnosed with COVID-19 were admitted to the hospital, of whom 80% were severely ill and critically ill patients, the oldest was 96 years old and the youngest was 13 years old. 14 patients underwent endotracheal intubation during treatment in the hospital.

14 patients with endotracheal intubation during treatment were aged between 36 and 90, years with a mean age of 70.15 ± 15.63, including ten patients older than 65 years, as shown in Table 1. 71.43% were males. Of note, only three patients had a clear history of contact in Wuhan epidemic area. The most common initial symptoms were fever (9, 64.28%), cough (4, 28.57%). One patient had no initial clinical symptoms and was diagnosed because of a positive SARS-CoV-2 test by viral nucleic acid testing. 64.29% of patients had the history of one or more underlying medical diseases: hypertension (8, 57.14%), diabetes (5, 35.71%), and cardiovascular and cerebrovascular disease (2, 14.29%). One of these patients was on long-term oral immunosuppressant-tacrolimus after liver transplantation. All patients had severe bilateral pulmonary infection on CT before endotracheal intubation.

**Table 1:** Baseline characteristics, major therapeutic measures and prognosis of patients with COVID-19 infection

Disease/Phenotype	Statistics of Intubated patients (14)
Age(years)	70.15 ± 15.63
Sex (M/F)	10/4
Wuhan residence or contact history	3
Hypertension	8
Diabetes	5
Malignant tumor	1
Cardiovascular and cerebrovascular diseases	2
COPD	0
Respiratory failure	1
Chronic kidney disease	3
Chronic liver disease	1
HIV	0
Tuberculosis	2
Main initial symptoms	
Fever	9
Cough	4
Diarrhea	0
Asymptomatic	1
Airway assessment: Mallampati class(≥ 3)	2
Chest CT before intubation	
Unilateral pneumonia	0
Bilateral pneumonia	14
ECMO	12
Lung transplantation	2
CRRT	2
Clinical outcome	
Discharged	14
Death	0

*COPD* Chronic obstructive pulmoriary disease, *HIV* Human immuno-deficiency virus, *CT* Computed tomography, *ECMO* Extracorporeal membrane oxygenation, *CRRT* Continuous renal replacement therapy

### Oxygen saturation and oxygenation index before, during, and after endotracheal intubation

The intubation process was smooth in 13 patients. The lowest SpO_2_ during intubation was 62.45% ± 13.4, and the hemodynamics during intubation was stable. All patients underwent endotracheal intubation through visual laryngoscope, and there were no complications related to endotracheal intubation. However, a patient with Mallampati class III and an indwelling gastric tube had poor mask ventilation, unsatisfactory glottic exposure. The lowest SpO_2_ decreased to 30% during intubation. After positive pressure ventilation therapy, SpO_2_ was still only about 60%. Fortunately, the second intubation was successful. In a review of 14 intubated patients, the mean arterial pressure during intubation was 82.49 mmHg ± 10.70.

Compared with before intubation, the PaO_2_ increased one day after intubation (59.52 mmHg ± 7.04 vs 113.77 mmHg ± 33.26 *p* < 0.001). Oxygenation index (PaO2·FiO2^−1^) and SpO_2_ also significantly increased compared with those before intubation (148.80 ± 42.25 vs 284.43 ± 60.17, *p* < 0.001; 88.44% ± 5.00 vs 96.48% ± 2.44, *p* < 0.001). PaCO_2_ was significantly improved after mechanical ventilation (33.60 mmHg ± 5.27 vs 43.91 mmHg ± 10.85 *p* = 0.011), as shown in Table 2.

**Table 2:** Comparison of oxygen partial pressure, oxygenation index, partial pressure of carbon dioxide and oxygen saturation before and after endotracheal intubation

Index	Before intubation	After intubation	*p*
PaO_2_	59.52 ± 7.04	113.77 ± 33.26	0.001
PaO2/FiO_2_	148.80 ± 42.25	284.43 ± 60.17	0.001
PaCO_2_	33.60 ± 5.27	43.91 ± 10.85	0.011
SPO_2_	88.44 ± 5.00	96.48 ± 2.44	0.001

### Adjuvant therapy and prognosis of patients with endotracheal intubation

**Fig. 1:**
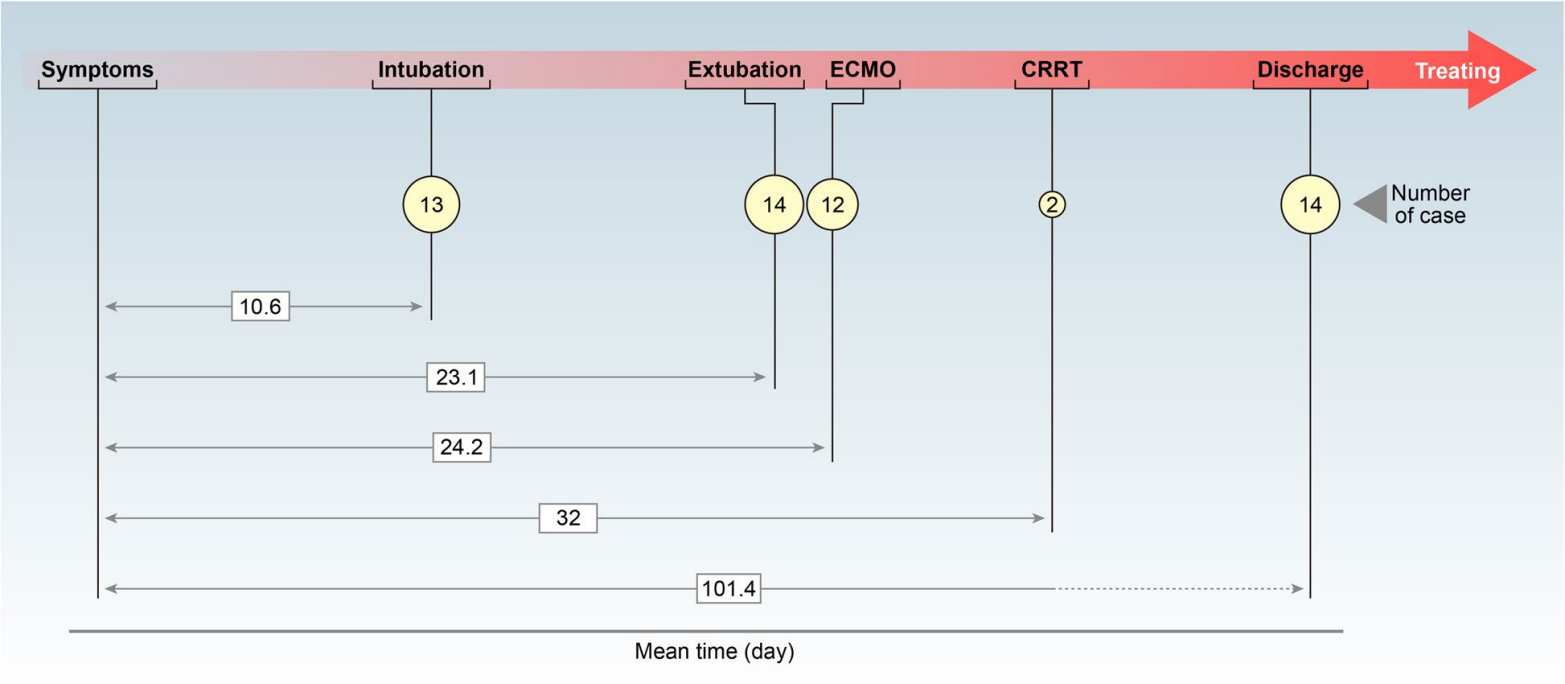
Timeline of treatment events after admission of COVID-19 patients. This study included 14 patients with tracheal intubation. Only the average time from symptom onset to tracheal intubation was counted in 13 patients. This is because one patient had no initial symptoms before being diagnosed with COVID-19. ECMO, Extra-Corporeal Membrane Oxygenation; CRRT, Continuous Renal Replacement Therapy

The mean time from onset of symptoms to endotracheal intubation was 10.6 days in the thirteen patients who completed endotracheal intubation (one patient had no initial symptoms), of whom two were re-intubated due to significant fluctuations in SpO_2_ after extubation, as shown in Fig.1. Twelve patients developed pulmonary infection rapidly, the oxygenation was not improved with standard lung ventilation strategies, so they were treated with ECMO, and the mean time from the onset of symptoms to ECMO was 24.2 days. Two patients received CRRT. Fourteen intubated patients had their endotracheal tube removed, with a mean time from onset of symptoms to extubation of 23.1 days, and were discharged uneventfully, with a mean time from onset to discharge of 101.4 days. No patients died during treatment.

Twelve intubated patients were treated with ECMO due to poor pulmonary function, and two of them underwent successful lung transplantation assisted by ECMO with good postoperative recovery [13]. All patients were discharged after satisfactory recovery. After 1 year of follow-up, all patients were alive and had no other respiratory and cardiovascular complications except the underlying disease.

## Discussion

Reasonable selection of intubation timing is particularly important, and no premature intervention or delay in intubation can reduce the oxygen debt of COVID-19 patients. In addition to the risk of hypoxia to the patient during intubation, there is also the risk of infection to the anesthesiologist directly exposed to SARS-CoV-2. Adequate and skilled intubation preparation and intubation are needed. The preparation of endotracheal intubation includes medical equipment, fasting and water-deprivation strategy, patient preparation (evaluating airway, etc.), self-protection of healthcare provider, etc. The intubation protocol is adjusted according to the status in actual operation based on the pre-prepared protocol. The mechanical ventilation should be implemented by sticking to the principles of optimizing oxygen and minimizing lung damage. The results of this retrospective study indicate that the critical COVID-19 patients who underwent endotracheal intubation at the hospital recovered satisfactorily. Based on the experience of COVID-19 diagnosis and treatment in the First Affiliated Hospital of Zhejiang University School and related research reports, we discussed the timing of tracheal intubation, intubation method, mechanical ventilation scheme and personal protection of medical staff.

Although some patients with COVID-19 have a low oxygenation index (< 100 mmHg), their clinical symptoms are mild. Whether such patients should be immediately intubated should be comprehensively considered in clinical setting. The most important is to pay attention to the progression of their underlying disease and comprehensively assess the patient's general status, compensatory ability and disease trend. Endotracheal intubation should be performed immediately when the patient develops hemodynamic instability and deteriorated state of consciousness. Endotracheal intubation criterion: when the patient shows evidence of persistent or progressively worsening respiratory failure and at least 2 of the following criteria are met: ① respiratory rate > 40 min^−1^; ② no signs of improvement in high respiratory load; ③ the presence of large amounts of airway secretions; ④ acidosis (pH < 7.35); ⑤ SpO2 < 90% for at least 5 min.

During the endotracheal intubation procedures, at least three health care anesthesiologists with more than five years of work experience are needed, with one respiratory therapist and one nurse additionally needed. The endotracheal intubation operation is scheduled in the negative pressure isolation ward. Anesthesiologists are required to be protected according to the Level 3 standard, shown in Table 3. However, inevitable shortcomings also follow, and the use of protective equipment and concerns about cross-infection can make the otherwise simple operation clumsy. The medical equipment and drug preparation before endotracheal intubation were similar to Professor Lingzhong Meng's regimen for first-line treatment of COVID-1 patients in Wuhan, as shown in Table 4 [12].

**Table 3:** COVID-19 Related personal protection management

Protection Level	Protective Equipment	Scope of application
Level I protection	Disposable surgical cap Disposable surgical mask Work uniform Disposable latex gloves or/and disposable isolation clothing if necessary	Pre-examination triage, general outpatient department
Level II protection	Disposable surgical cap Medical protective mask (N95) · Work uniform Disposable medical protective uniform Disposable latex gloves Goggles	Fever outpatient department · Isolation ward area (including isolated intensive ICU) Non-respiratory specimen examination of suspected/confirmed patients Imaging examination of suspected/ confirmed patients Cleaning of surgical instruments used with suspected/confirmed patients
Level III protection	Disposable surgical cap Medical protective mask (N95) Work uniform Disposable medical protective uniform Disposable latex gloves Full-face respiratory protective devices or powered air-purifying respirator	When the staff performs operations such as tracheal intubation, tracheotomy, bronchofibroscope, gastroenterological endoscope, etc., during which, the suspected/confirmed patients may spray or splash respiratory secretions or body fluids/blood When the staff performs surgery and autopsy for confirmed/suspected patients When the staff carries out NAT for COVID-19

Notes: 1. All staff at the healthcare facilities must wear medical surgical masks; 2. All staff working in the emergency department, outpatient department of infectious diseases, outpatient department of respiratory care, department of stomatology or endoscopic examination room (such as gastrointestinal endoscopy, bronchofibroscopy, laryngoscopy, etc.) must upgrade their surgical masks to medical protective masks (N95) based on Level I protection; 3. Staff must wear a protective face screen based on Level II protection while collecting respiratory specimens from suspected/confirmed patients

**Table 4:** Endotracheal intubation medication and equipment preparation

Components	Action	Backup Plan
O:Oxygen	Ensure an adequate supply of oxygen is available	Ensure a separate, full oxygen tank is available in the room
H:Helpers	Identify and ensure helpers are readily available	Clearly understand how to obtain the needed help
M:Monitor	Ensure pulse oximetry, electrocardiography, and noninvasive blood pressure monitors are functional	Ensure backup monitors are readily available, at least outside of the room
S:Suction	Ensure suction is functional and readily available	Ensure a separate (may be portable) suction is available
M:Machine	Ensure an anesthesia machine or an ICU ventilator is functional and ready to go	Ensure a bag-mask system (*e.g.,* Ambu bag) capable of positive-pressure ventilation is readily available
A:Airway supplies	Ensure the video laryngoscope (e.g., GlideScope) is functional and have a direct laryngoscope as a backup	Have a difficult airway cart in the room if a difficult airway is anticipated; otherwise, it should be readily available but outside of the room
I:Intravenous access	Flush and ensure functional intravenous access	Have the supplies readily available in case a new access site is needed
D:Drugs	Have all drugs for sedation, anesthesia induction and muscle relaxation and different vasoactive drugs prepared	Have a drug tray based on the same standards for OR and ICU settings

*ICU* Intensive care unit, *OR* Operating room

Compared with common laryngoscope, video laryngoscope can not only expose glottis in a better way, but also increase the distance between anesthesiologist and patient airway, thus reducing the risk of cross infection in anesthesiologists. Fiberoptic bronchoscopy is only used when laryngoscopic intubation fails. Because fiberoptic bronchoscopy has a long operation time, it will increase the time of no oxygen supply to the patient. To prevent cross-contamination between patients, devices such as disposable laryngoscope blade can be used.

To prevent additional pulmonary infection in COVID-19 patients caused by aspirating gastric contents, consulting with ICU physicians regarding the duration of fasting and water-deprivation was needed. In addition, before an endotracheal intubation, the airway should be effectively assessed.

The retrospective study found that COVID-19 patients had a very poor ability to tolerate hypoxia for a short time during intubation operation. In 14 patients with COVID-19, their lowest oxygen saturation during intubation was 62.45% ± 13.4. Therefore, preoxygenation before intubation appears particularly important. Most COVID-19 patients treated in the ICU of the hospital were given high-flow nasal cannula oxygen therapy or non-invasive positive pressure ventilation. If a patient has previously received nasal cannula high-flow oxygen therapy, it can be continued during endotracheal intubation after 5 min of positive pressure ventilation with 100% oxygen via mask. If the patient is treated with non-invasive positive pressure ventilation, it can be continued for 5 min before intubation, in combination with high-flow nasal cannula oxygen therapy during the intubation. Continuous use of nasal cannula during tracheal intubation can increase oxygen supply, but it also increases aerosol particles production.

Endotracheal intubation is performed after safe and effective induction of anesthesia. The choice of anesthetic drugs is based on the principle of reducing patient oxygen consumption and circulatory fluctuations. Oxygen consumption can also be reduced by reducing anxiety in patients using 1—2 mg of midazolam. For patients with stable hemodynamics, induction is performed using propofol (1—1.5 ml·kg^−1^). Otherwise, etomidate (0.15—0.3 ml·kg^−1^) is a better choice. However, the incidence of etomidate-induced muscle tremor is high, which will aggravate oxygen consumption in patients. Therefore, its use should be avoided as much as possible. For the aforementioned difficult airway, we do not recommend the use of muscle relaxants. For patients with a non-difficult airway, the use of muscle relaxants can inhibit the cough reflex, thereby preventing viral particles from splashing on healthcare providers. Vecuronium (0.1—0.13 ml·kg^−1^) is available. Opioids can reduce hemodynamic fluctuations in patients with endotracheal intubation, but opioids can cause choking reactions and increase aerosol particle production. Intravenous injection and topical airway spraying of lidocaine can reduce this airway responsiveness. Vasoactive and cardiotonic drugs require routine backup.

In patients with a difficult airway, rapid induction is recommended. If difficult mask ventilation after induction is predicted, intubation on the basis of retention of spontaneous breathing is recommended. Patients with COVID-19 not only have poor lung function but also have increased airway secretions. Hypoxia plus secretion greatly increases the risk of laryngospasm. Sputum suction before tracheal intubation and tracheal surface anesthesia can reduce the incidence of cough reflex and laryngospasm.

Level III protection with a positive pressure head cover is recommended, which could prevent vapor generated by the head cover and goggles from blocking the field of vision. Since the medical staff wear a head cover, it is not feasible to assess the position of the tracheal tube by auscultating the patient's breath sounds. Observing endotracheal tube vapor is still feasible. After connection to the ventilator, the thoracic fluctuation and carbon dioxide waveform are observed to determine the position of the endotracheal tube. The correct position and depth of the endotracheal tube should be ensured at all times to avoid pulling of the tube and to prevent unplanned extubation. Attempts should be made to ensure the patency of artificial airway, prevent sputum and gastric contents from being sucked into the lungs and promote the discharge of airway secretions. If an endotracheal tube with a subglottic suction feature is used, intermittent suction can be performed after endotracheal intubation. Disconnection of the ventilator from the endotracheal tube should be avoided at all times.

The risk of ventilator-associated lung injury should be minimized during mechanical ventilation. To avoid man–machine confrontation, patients should be given appropriate sedation and analgesia. If necessary, muscle relaxants should be used, especially in the early stages of ventilator use. Conservative oxygen therapy strategy and conservative fluid management strategy should be adopted. Health care providers should adjust the PEEP levels reasonably and perform titration of PEEP according to patient response. Patients with obesity, increased intra-abdominal pressure, and pleural effusion may require higher PEEP levels and plateau pressure. If conditions permit (with enough guardian) and under the premise of ensuring safety, prone position ventilation is implemented for the patients with oxygenation index of less than 100. It is recommended to maintain the prone position for 12 ~ 16 h·d^−1^.

**Fig. 2:**
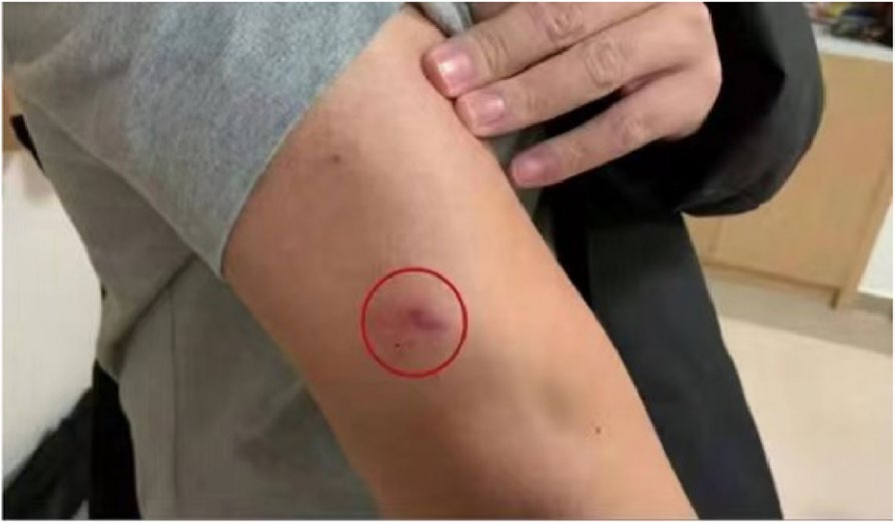
A doctor bitten by a COVID-19 patient during extubation

When a doctor extubated for a COVID-19 patient in the (Intensive care unit) ICU of the hospital, his arm was bitten by the patient, as shown in Fig. 2. Fortunately, the patient’s viral nucleic acid detection was negative, and the doctor was in personal protective equipment, which was not broken after the biting. Wearing personal protective equipment is important at the time of extubation. Extubation can be dangerous if the patient is irritable with the delirium symptoms. The use of the sedative agents prior to extubation is recommended to prevent irritability and delirium. Dexmedetomidine (0.4 mcg·kg^−1^·h^−1^) or remifentanil (1 to 4 ng·ml^−1^ target blood concentration) have been recommended.

## Conclusions

Reasonable selection of intubation timing is particularly important. It is crucial to increase the patient's oxygen supply and reduce oxygen consumption as much as possible during endotracheal intubation. In addition, the personal protective measures of medical personnel participating in treatment should be scientific and standardized.

## Data Availability

The datasets used or analysed during the current study are available from the corresponding author on reasonable request.

## Abbreviations

COVID-19: Coronavirus disease-19
ECMO: Extra-corporeal membrane oxygenation
ARDS: Acute respiratory distress syndrome
COPD: Chronic obstructive pulmoriary disease
HIV: Human immuno-deficiency virus
CT: Computed tomography
CRRT: Continuous renal replacement therapy
ICU: Intensive care unit
